# Quality of Life, Needs and Fears of Mothers of Children with Disabilities in Saudi Arabia during the COVID-19 Lockdown

**DOI:** 10.3390/ijerph182111442

**Published:** 2021-10-30

**Authors:** Nisreen Al Awaji, Monira Aldhahi, Shahnaz Akil, Salwa Awad, Eman Mortada

**Affiliations:** 1 Department of Health Communication Sciences, College of Health and Rehabilitation Sciences, Princess Nourah Bint Abdulrahman University, Riyadh 84428, Saudi Arabia; 2 Department of Rehabilitation Sciences, College of Health and Rehabilitation Sciences, Princess Nourah Bint Abdulrahman University, Riyadh 84428, Saudi Arabia; Mialdhahi@pnu.edu.sa (M.A.); SASAhmed@pnu.edu.sa (S.A.); 3 Department of Laboratory Medicine, Clinical Physiology, Karolinska Institute, SE-14186 Stockholm, Sweden; Shahnaz_161@hotmail.com; 4 Department of Health Sciences, College of Health and Rehabilitation Sciences, Princess Nourah Bint Abdulrahman University, Riyadh 84428, Saudi Arabia; EMMortada@pnu.edu.sa; 5 Community, Environmental, and Occupational Medicine Department, Faculty of Medicine, Zagazig University, Zagazig 44519, Egypt

**Keywords:** COVID-19, quality of life, mothers, children with disabilities, support, Saudi Arabia

## Abstract

Substantial changes in life dynamics resulting from the outbreak of the coronavirus disease 2019 (COVID-19) could have an impact on the quality of life (QoL) of mothers of children with and without disabilities. This study compared the quality of life (QoL) of mothers of children with disabilities (MCD) to the QoL of mothers of children without disabilities (CON) in Saudi Arabia during COVID-19 lockdown. It explored mothers’ concerns and the type of support they need during the quarantine. A comparative cross-sectional study was conducted during the lockdown. An online questionnaire was distributed to mothers raising children with and without disabilities in Saudi Arabia. A total of 340 mothers participated in the study by completing the survey: 93 MCD and 247 CON. The QoL of MCD and CON was assessed using the WHOQOL-BREF questionnaire. Furthermore, detailed information was provided by the mothers regarding their needs and concerns during the lockdown. The results of the study revealed that the overall QoL was significantly higher in the CON group, compared to the MCD group, during the COVID-19 lockdown. The social well-being and environmental well-being reported by MCD were significantly lower on the total scale of the WHOQOL-BREF than those reported by the CON group. The comparison between the two groups revealed significant differences in the support required by mothers during the COVID-19 pandemic: a higher percentage of MCD needed emotional and psychological support, especially from family members. The major concerns reported by MCD were the deterioration of their children’s medical conditions and the lack of medical supplies during the lockdown.

## 1. Introduction

The coronavirus disease 2019 (COVID-19) is the most recent highly contagious human disease. It is derived from the severe acute respiratory syndrome coronavirus 2 (SARS-CoV-2) [1]. As a highly communicable novel disease with an exponential growth rate, COVID-19 gained global attention after being officially declared a pandemic by the World Health Organization (WHO) on 11 March 2020 [2]. The emergence of COVID-19 has overwhelmed health systems across the globe, generating alarming death rates in many countries worldwide [3]. As of 28 June 2020, more than 9.88 million cases and more than 490,000 deaths have been reported globally. The rapidly escalating COVID-19 crisis has created a massive threat to humanity, which could potentially have profound long-term impacts on public health, as well as detrimental effects on psychological health and economic, social and religious life. In response to this threat, governments worldwide have complied with WHO guidelines and initiated social distancing and lockdown measures to restrain the spread of the disease, which still has no officially confirmed cure [4].

Saudi Arabia implemented strict precautionary measures on 1 February 2020. This included suspending all flights from China before any cases were confirmed in the country. Thereafter, all incoming and outgoing flights were banned. On Monday, 9 March 2020, all educational systems in Saudi Arabia were suspended temporarily and replaced by remote learning using virtual classes. This was followed by a nationwide curfew under which leaving home was only permissible for adults, during specific times, in necessary cases. Most hospitals also implemented measures to limit exposure to patients, visitors and staff, postponing non-urgent surgeries and visits to outpatient clinics.

Different studies have reported the negative impact of quarantine on people’s physical and psychological wellbeing [5,6], which may consequently affect their QoL. The COVID-19 lockdown has negatively impacted people’s mental health [7], and parents of children with disabilities are particularly vulnerable to such problems, given that their QoL is relatively low even in the absence of a pandemic [8]. Generally, children with disabilities need help with their daily activities, and due to the frequent care they require [9], their parents’ QoL may be compromised by increased physical demands, lack of sleep, financial burdens and difficulty taking family holidays [10]. Restrictions related to COVID-19 may act as additional stressors to children with disabilities and their families, as certain routines that were embedded in their lives have now changed [11]. Failure to access formal support provided by schools, respite services and healthcare professionals, as well as a lack of informal support from family members and friends, may place particular burdens on MCD. 

The QoL of mothers who care for children with disabilities is an area of concern that needs to be investigated in depth in Saudi Arabia. Given the cultural tendency, women in this country are responsible for taking care of the house and all family members who live in the house, which sometimes includes grandparents [12]. Therefore, the responsibilities of a mother in Saudi Arabia may become extreme if the family has a child with a disability and if the mother works outside the home [11]. For a working mother, levels of stress and worry may increase when a lockdown forces her to work from home in the presence of her children. This could add to the difficulty of maintaining a healthy balance between various duties, which may negatively influence a mother’s QoL. Thus, there is a clear need to evaluate the QoL of MCD compared to CON during the COVID-19 lockdown. Saudi Arabia presents a useful case study to examine these groups during the COVID-19 lockdown. Given that most children are dependent on their mothers, the lockdown has likely created substantial modifications to the dynamics between children and their mothers. Therefore, the aim of this study was to compare the QoL between mothers of children with disabilities (MCD) and mothers of children without disabilities (comparison group: CON) in Saudi Arabia during the COVID-19 lockdown. The study also aimed to determine the concerns MCD had and the types of support they needed during lockdowns.

## 2. Materials and Methods

### 2.1. Study Design and Population

A comparative cross-sectional study was performed in May and June 2020, during the COVID-19 lockdown in Saudi Arabia. A link to an online questionnaire was distributed by all members of this study’s research team through different types of social media (Twitter, Facebook and WhatsApp) to different groups of mothers currently living in the country (students, faculty, healthcare workers, friends, members of Saudi associations for children with disabilities) using the snowball sampling technique. The inclusion criteria were as follows: agreement to participate in the study, current residence inside Saudi Arabia and being a mother of a child ≤ 18 years. 

The calculation of the target sample size (n) was based on the following equation: n = [DEFF × Np(1 − p)]/[(d2/Z21 − α/2 × (N − 1) + p × (1 − p)], where “DEFF” (design effect for cluster surveys) was 1; “N” (population size) was 667,280 [13]; “p” (hypothesized % frequency of outcome factor in the population) was 33%+/−5, and “d” (confidence limits as % of 100) was 5%. An open-source calculator (OpenEpi) was used to calculate the targeted sample size which was found to be 340 responses (Table S1). A total of 496 responses were received, of which 156 respondents were excluded either because they did not meet the inclusion criteria or because of incomplete responses. Therefore, a total of 340 respondents were included in this study, which were further dichotomized into 93 in the MCD group and 247 in the CON group. Based on the final included sample, the following prevalence rates of disability were reported: 24% of mothers had children with autism; 23% had children with a physical impairment; 19% had children with Down syndrome, 6.5% had children with growth retardation; 6.5% had children with thyroid cancer, kidney problems or brain hypoxia; 7.5% had children with hearing impairment; 7.5% had children with ADHD; 3% had children with language delay; 2% had children with sensory impairment; and 1% had children with mental impairment.

### 2.2. Questionnaire

A questionnaire in Arabic was generated using an online survey system (SurveyMonkey); it consisted of three parts: sociodemographic characteristics, QoL among mothers and mothers’ needs and fears.

#### 2.2.1. Sociodemographic Characteristics

A total of 15 questions related to the mother’s personal information were asked, including age (18–25, 26–35, 36–45, 46–55, 56–60, more than 60 years), nationality (Saudi, gulf country national, Egyptian, Sudanese, Syrian, Palestinian, Moroccan, Yemeni, other), gender, educational level (elementary school, secondary school, higher education, postgraduate studies), marital status (married, separated, divorced, widowed), general health status (absence of health problems, presence of health problems), occupation (housewife, employed in the government sector, employed in the private sector, unemployed), general psychological status (absence of psychological illness, presence of psychological illness), perceived economic status (low input, moderate input, high input) and number of children between 0 and 18 years old (zero, one, two, three, four, five). If the mother had children with disabilities, she also answered questions related to the number of children with disabilities (free response) and their gender, whether the pregnancy was term or preterm for each of those children, and the children’s types of disability (autism spectrum disorder, hyperactivity and distraction, language delay, Down’s syndrome, epilepsy, delayed overall growth, hearing impairment, physical disability, mental difficulties, speech problems, sensory disturbance, vision problems/blindness, other: please specify). More than one type of disability could be selected.

#### 2.2.2. Quality of Life among Mothers

Questions were adapted from the standardized Arabic, brief version of the World Health Organization Quality of Life questionnaire (WHOQOL-BREF) [14]. The survey was modified by adding the words “in the presence of COVID-19 lockdown" to the question stem of each domain to allow the measurement of the participants’ perception of their QoL during the COVID-19 lockdown. One item related to sexual health was removed from the standardized 26-item WHOQOL-BREF due to cultural sensitivity. 

In total, 25 items from the standardized WHOQOL-BREF were used in this study, with seven items assessing physical health (Domain 1)**,** six items assessing psychological health (Domain 2)**,** two items assessing social relations (Domain 3), and eight items assessing environment (Domain 4) [15]. The remaining two items measured the participants’ overall QoL and level of health satisfaction. The participants rated their answers using a scale rating agreement (1 = not at all, 2 = a little, 3 = a moderate amount, 4 = mostly and 5 = completely) or a scale rating satisfaction (Likert scale) that ranged from very dissatisfied (1) to very satisfied (5) with a neutral option in the middle (neither satisfied nor dissatisfied). Cronbach’s Alpha revealed adequate reliability for the WHOQOL-BREF and its subscales for the overall sample and for both studied groups, as can be seen in Appendix 2.

#### 2.2.3. Mothers’ Needs and Fears 

Two open-ended questions were asked related to mothers’ concerns during the COVID-19 lockdown and the type of support they needed for their children. The two questions were phrased as follows: “With the suspension of access to various curative services, what are your greatest needs and concerns related to your child during the COVID-19 lockdown?” and “What kind of support did you need and see as beneficial to you and your child during the COVID-19 lockdown?” The responses to these two questions were numerically coded. The expressed concerns, fears and required supports of MCD were compared to those of the CON group.

### 2.3. Ethical Considerations

The study was ethically approved by the Institutional Review Board (IRB) at Princess Nourah Bint Abdulrahman University (20-0191), Riyadh, Saudi Arabia. A consent question was added to the survey to ensure that the survey responders agreed to participate in the study. Complete information about the study and the email of the principal investigator were also provided in the survey description.

### 2.4. Statistical Analysis

The software SPSS Version 20 was used for statistical analysis. Data were expressed as frequencies and percentages for nominal variables, mean ± SD for continuous normally distributed variables and median and interquartile range for individual items using Likert scale responses. A *p*-value ≤ 0.05 was considered statistically significant. Fisher’s exact test was used to compare the distribution between study groups in the mothers’ needs and fears. For the part of the questionnaire related to QoL, the Kolmogorov–Smirnov test was used to assess the normality of the distribution of raw scores of outcome variables (WHOQOL-BREF and its subscales) among the studied groups. An independent Student’s t-test was used for the normally distributed outcome, and the Mann–Whitney test was used when comparing the median scores for overall QoL and overall health satisfaction. Moreover, appropriate effect size was calculated with all used statistical tests. Multiple regression analysis was used for identity-independent factors that may significantly predict the outcome variables (WHOQOL-BREF). 

## 3. Results

### 3.1. Sociodemographic Characteristics

Table 1 displays sociodemographic and health-related characteristics of the respondents. A slight majority of the participants were housewives (174, 51.2%) rather than working mothers (166, 48.8%). A total of 229 (68%) of the mothers were ≥36 years. Most of the participants (74%) had a moderate income. When comparing sociodemographic characteristics and the health status of the respondents between the two groups of mothers (MCD and CON), a significant difference in age was found between the study groups (χ^2^ = 7.79, *p* = 0.05), as displayed in Table 1.

College and postgraduate education levels were around 15 and 38 times higher, respectively, among mothers without children with disabilities (95% CI: 4.4–56.9; 95% CI: 8.2–100.9 respectively; *p* < 0.001). Similarly, income levels were significantly different (χ^2^ = 42.07, *p* < 0.001), as moderate- and high-income levels were significantly higher, respectively, among mothers without children with disabilities as compared to MCD (*p* < 0.001). The majority of the working mothers (56%) reported having children without disability whereas only 30% of the mothers of children with disabilities were employed (*p* < 0.001). As presented in Table 1, physical health problems were not significantly different between the groups.

### 3.2. Quality of Life among Mothers

#### 3.2.1. Comparison of Quality of Life between Groups 

**Figure 1:**
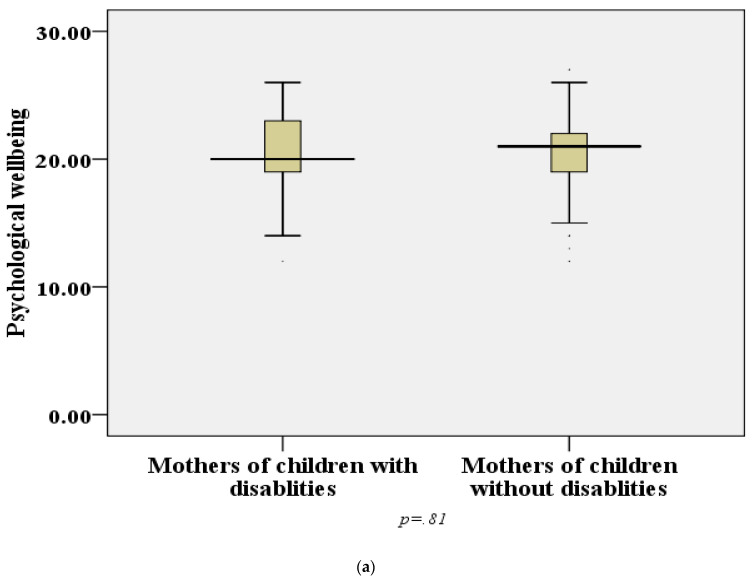

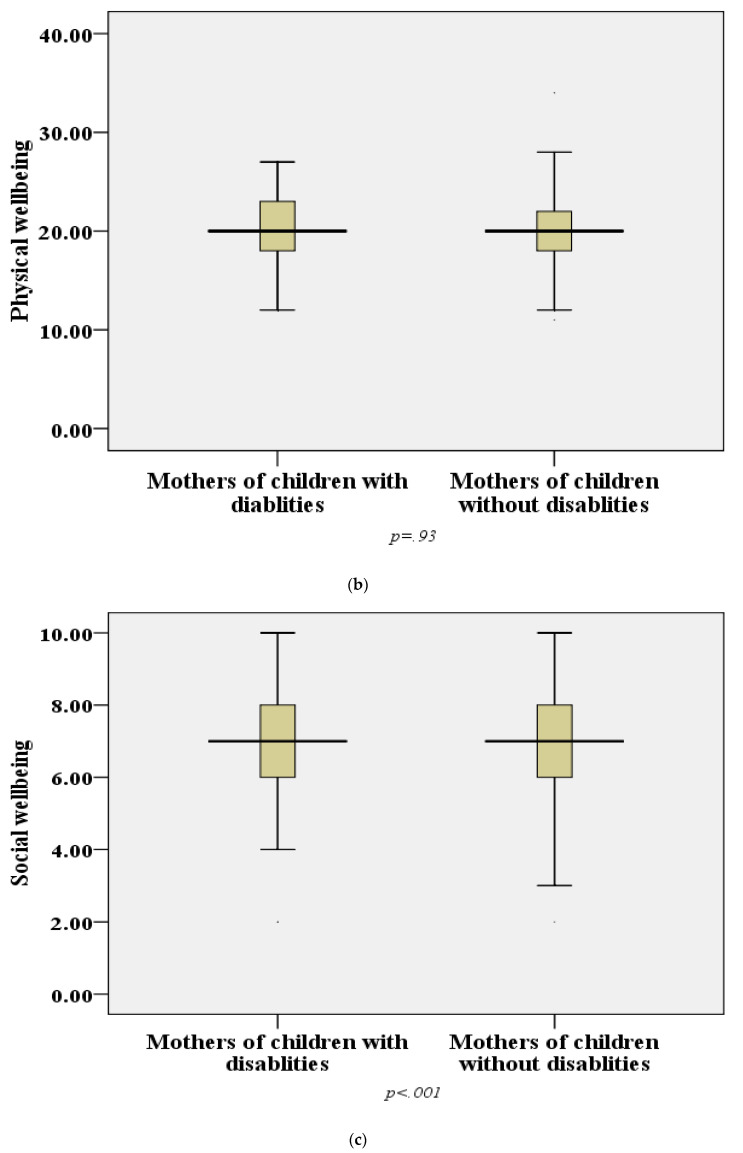

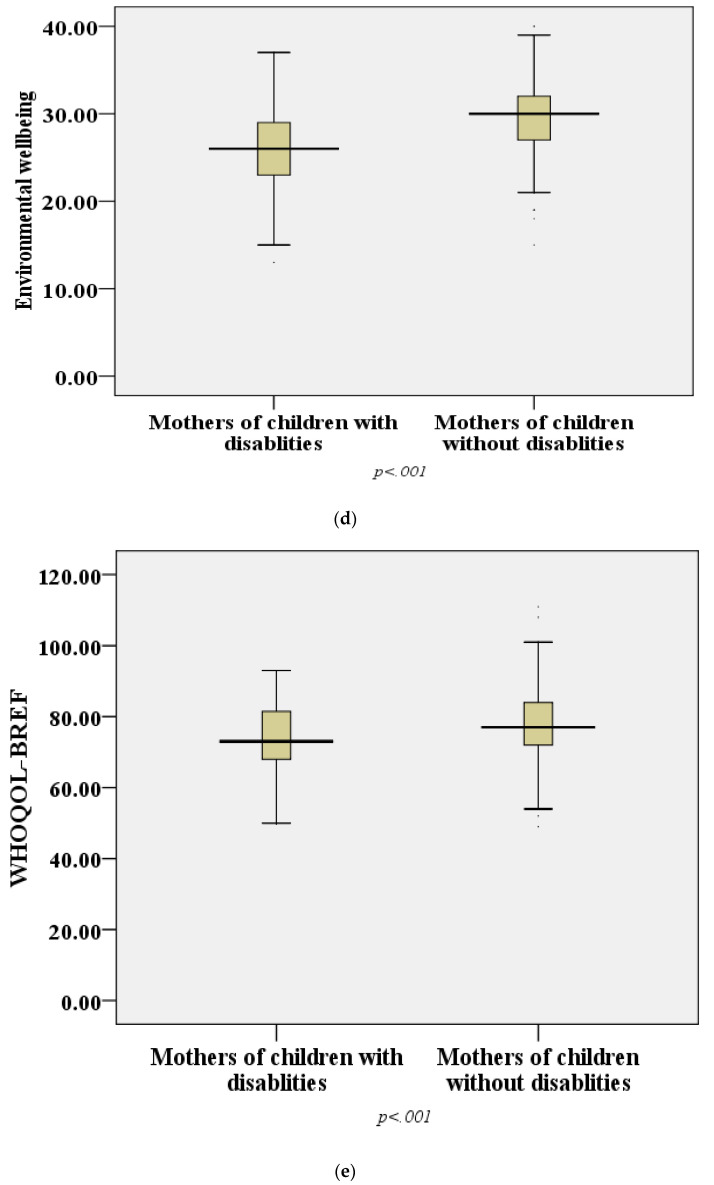
WHOQOL-BREF subscales among mothers of children with disabilities and mothers of children without disability during COVID-19 lockdown. (**a**) psychological health; (**b**) physical health domain; (**c**) social relations; (**d**) environmental health; (**e**) total QoL scores.

A Mann–Whitney test indicated that overall QoL was significantly higher in the control group (mean rank of MCD 136 vs. CON 184, *p* < 0.001, r = 0.25). The median scores for the five domains of WHOQOL-BREF are shown in Figure 1. Social well-being and environmental well-being reported by the MCD were significantly lower (*p* < 0.29) on the total scale than those of the CON group (Figure 1). It can be seen in Table 2 that there is no significant difference (*p* = 0.29) between the groups in the reported level of health satisfaction. 

Items assessing the environmental well-being domain of the WHOQOL-BREF are reported in Table 3. Items regarding the environmental well-being of mothers of children with disabilities were significantly different in six aspects: financial needs (*p* < 0.001, effect size = 0.3); availability of information about COVID 19 in day-to-day life (*p* < 0.001, effect size = 0.20); opportunity for leisure activities during the COVID-19 period (*p* < 0.001, effect size = 0.21); satisfaction with living place, access to health services, and transportation (*p* = 0.001, effect size = 0.18), (*p* < 0.001, effect size = 0.21)*,* and (*p* = 0.002, effect size = 0.9), respectively.

#### 3.2.2. Predictors of Quality of Life 

Table 4 presents the multiple linear regression, a model including age, nationality, education, occupation, income, marital status, children with disabilities, health problems and mental problems. It has been demonstrated that age (β = 0.10, *p* = 0.04) and children with disabilities (β = 0.17, *p* = 0.003) explained a relatively small amount of overall variance in QoL (R^2^ = 0.103, F = 4.2, *p* < 0.001). However, occupation (*p* = 0.17), income (*p* = 0.18) and mental problems (*p* = 0.07) did not explain the variations in QoL. Table 5 displays group-specific correlation analysis, which showed the lack of a significant association between QoL and sociodemographic characteristics among mother of children with disabilities (Table 5A). The lack of an association between QoL and sociodemographic variables excludes the confounding effect of these variables on QoL and the fact that other variables may drive these changes in the QoL. Among mothers of children without disabilities, there was a significant relationship between QoL and occupation status, income and age (Table 5B).

#### 3.2.3. Mothers’ Needs and Fears 

Investigating the factors that contributed to fears among mothers, it was demonstrated that a high percentage of mothers’ fears were related to the infection of their child with COVID-19 (MCD 32%; CON 44%). The deterioration of their children’s medical conditions and lack of medical supplies during the lockdown period were major concerns among mother of children with disabilities (Table 6A). In terms of the fears among mother of children without disabilities, it has been determined that exposure of a child to home accidents and emergencies and a lack of access to timely medical care due to the lockdown were primary concerns.

In terms of the reported needs of mothers in the MCD group and the CON group, the item of emotional, psychological and family support showed the highest percentage among mothers in both groups (20%) and should be embraced (Table 6B). In line with that finding, one item in the psychological domain of the WHOQOL-BREF related to negative feelings, such as a blue mood, despair, anxiety and depression, significantly differed between the groups of mothers (2.97 ± 1.06 vs. 2.51 ± 0.94, t (264) = 3.88, *p* < 0.001, d^t^ = 0.5). The mothers’ needs presented in Table 6 revealed that entertainment was a demand among mothers who have children without disabilities (18%) and a lower priority among mothers who have children with disabilities (6%).

## 4. Discussion

The COVID-19 pandemic has forced the implementation of several precautionary measures worldwide, which may cause additional burdens on vulnerable groups in society, including MCD. The overarching aim of the current study was to examine the QoL of MCD and compare the results to CON in Saudi Arabia during the COVID-19 lockdown period. The study showed evidence of an overall lower QoL reported by MCD compared to the QoL reported by mothers in the CON group. Analysis of the WHOQOL-BREF scale showed a significantly lower QoL for the MCD group than for the CON group in terms of social and environmental well-being and leisure activities. Additionally, the MCD described concerns that arose during the COVID-19 lockdown, which included the deterioration of children’s medical conditions and a lack of access to medical supplies.

### 4.1. Quality of Life

A study conducted among Saudi women, by comparing pre-pandemic data with lockdown data, disclosed the consequences of the lockdown on women’s lifestyle factors including weight change, sleep, mental health, physical activity and dietary habits [16]. It revealed that the high risk of weight gain during lockdown was associated with moderate mental stress, whether it was developed before or increased during lockdown, which could be related to increased food desires that lead to unnecessary eating, particularly among young women.

In the current study, the overall QoL rating was significantly higher in the CON group than in the MCD group. This is in line with the findings of previous studies that show that the characteristics of a major caregiver’s life may be influenced by a number of factors related to a child’s disability [17], including excessive physical demands, irregular sleep, difficulty taking family holidays, financial limitations and inadequate family and social support [18].

The well-being of parents of children with disabilities is a blend of multi-factorial clusters in three main categories: (1) the individual characteristics of the child, such as health, age and gender; (2) parents’ personal judgments with regard to stress levels and belief in their individual ability to face the difficulties of rearing a child with a disability; and (3) environmental/social conditions, such as a lack of financial support, family resources, care and provision of services [19]. A caring environment is likely to reduce parents’ stress levels and reinforce their positive functioning [2,20,21]

In this study, QoL is divided into four domains: physical, psychological, social and environmental. It is worth mentioning that the two groups of mothers did not differ in their physical and psychological well-being. This contrasts with previous studies that have reported that the physical and psychological well-being of MCD is lower than that of the CON group [22,23]. The same finding, however, was also reported by Alwhaibi et al. [24], who found no difference in psychological satisfaction between the two groups. The same study conducted by Alwhaibi et al. [24], explored the family dimension of QoL with regard to family health, children, family happiness, spouse and emotional support from the family; it found no significant difference between mothers of children with or without disabilities. Both groups of Saudi mothers attributed high importance to families, which confirms the respect felt in families that are cohesively united into groups. Such tight-knit families are often extended families that devotedly attend and support members who are mothers [25]. The presence of children with disabilities usually causes familial disturbances due to the additional demands of care these children require [26]. However, married parents remain within the family context pay attention to their children and keep the ties that are treasured by the Saudi community irrespective of the children’s disability conditions. 

On the other hand, the level of social and environmental well-being reported in the MCD group was significantly lower (*p* < 0.001) than in the CON group. It can be inferred that this might be related to the responsibilities that MCD bear in attend to their children’s special needs, which leave them with less time to conduct their own personal life as they desire. Therefore, their environmental and social well-being is disrupted, and their QoL in these domains is decreased. 

The lack of social relationships reported by MCD during the lockdown negatively affected their QoL. Prior to the lockdown, MCD found it helpful to mingle with other people to exchange thoughts and ideas about the causes of stress and how to cope with them. Other sources of support prior to the lockdown were the educational and specialized institutions where most children with disabilities were taken on a regular basis, which helped mothers find time to get involved in other activities. This relieved mothers from the utilitarian burden they face in taking care of their children and the challenges of intensive, tough, sometimes lifelong caregiving. Support can be physical, financial, or emotional, such as care and encouragement from close individuals [27]. A study illustrated that Saudi MCD are in need of additional social support and specialized help to boost adaptive coping behaviors that can empower them to face the worries of raising a child with a disability [24].

Saudi families of children with disabilities receive different kinds of assistance from the Ministry of Labor and Social Development (MLSD), such as access to rehabilitation centers and annual funds to cover expenses related to a child’s disability [12,28]. This practice needs to be nurtured among families, particularly MCD, so that governmental services can be fully utilized to provide education and training of children with disabilities, which can be considered social and environmental support.

In the present study, a comparison of the items regarding the environmental well-being of MCD and the CON group performed during the COVID-19 lockdown showed a significant difference between the two groups: MCD reported greater levels of worry over not having enough money to cover the demands of their children. They might need to secure personal protective equipment, home-care services and rehabilitation services. The lack of care and funds coupled with increased demands due to the lockdown tended to induce an accumulative effect that may impact the physical and mental health of MCD. There was also a significant difference between the MCD group and the CON group concerning the availability of day-to-day information about COVID-19. Here, it is vital to mention that there was excessive information presented through social media that was unnecessary and, consequently, left a negative effect on the population. The data that was most useful to MCD had to do with advice and information on preventive procedures provided by the Ministry of Health and professional healthcare providers.

Leisure activities have also been severely affected by the lockdown. There was a significant difference between MCD and the CON group concerning their feelings towards the lack of activities. A low level of exercise for children with disabilities can lead to weight gain and loss of skills acquired prior to the lockdown. The MCD also expressed dissatisfaction with the conditions of their living place. It was essential for MCD to have recreation time for their children as the unexpected lack of mobility could have an unfavorable effect on their children’s health. 

### 4.2. Required Support for Mothers of Children with Disabilities

Emotional and psychological support was reported by most of the mothers in the MCD group (26%) as the most important type of support that would, in their opinion, help them and their children. The psychological domain of the WHOQOL-BREF, which includes one of the items related to negative feelings, such as a blue mood, despair, anxiety and depression, illuminated a significant difference between the MCD group (2.97 ± 1.06) and the CON group (2.51 ± 0.94; *p* < 0.001). The findings of this item in the current study should be taken seriously, as a previous study demonstrated that a lack of psychological support for a period of 18 months was linked with a high level of depressive signs, negative affect and declined optimism [29]. Furthermore, for parents of children with ASD, lower levels of social support have been associated with negative outcomes, such as an increase in somatic symptoms [30]. Caregivers with a frail and deteriorated psychological well-being can also negatively affect children’s QoL [31].

The second-most helpful support reported by MCD is family support. Weak ties between family members and friends escalate psychological discomfort and increase the burden placed on MCD. As a result, they feel lonely, abandoned, socially isolated, rejected and locked away from their social circle. This leaves them with more unfulfilled requirements in different aspects of their lives [29,30]. MCD are certainly enduring a difficult time during the current lockdown period. During normal circumstances, their suffering can be alleviated by getting involved in family gatherings, particularly with extended family members, and attending care centers, schools and recreation hubs. In addition, MCD are often considered the primary caregivers for the whole family, which makes it more difficult for them to cater to their own well-being and happiness. Spiritual support is also among the needs expressed by MCD in the present study. Empirical evidence suggests that faith and spirituality are significant coping mechanisms that underpin well-being [32]. Faith and spirituality are also driving forces that encourage MCD to overcome everyday difficulties and provide meaning to the situations faced [33].

Exploring the factors that increase worry among mothers during the COVID-19 lockdown, the results reveal that a high percentage of the fears (18%) stated by MCD were attributed to the deterioration of their child’s medical condition and lack of medical supplies during the lockdown period. Assessing the extent of the constraints that prevent the mothers from obtaining medical treatment during the lockdown, we see that MCD tend to report a moderate level of restriction, higher than that of the CON group. Medical services are an essential demand of MCD due to their children’s medical conditions. Mothers need consultations with primary medical doctors regarding the condition of their child or medical supplies. It is important to have enough medicine available during the lockdown. Some conditions require continuous medicine-taking with no interruption. Ceasing medication adherence may worsen an individual’s condition. Moreover, four MCD expressed worries about difficulties that they might face because they were not able to access healthcare services prior to the outbreak of the virus. They also expressed concerns about the effect of limited access to healthcare on the health of their children.

## 5. Conclusions

This study investigated the QoL, needs and fears of mothers of children with disabilities and mothers of children without disabilities in Saudi Arabia. Results indicated that MCD face more challenges than do mothers of children without disabilities. This is expressed by lower QoL scores in the social relationships and environmental domains. The mothers were also asked about the type of supports needed during the COVID-19 pandemic. Frequently mentioned required supports included emotional and psychological support as well as family support. 

Social support provided by family members and friends is vital in helping MCD in Saudi Arabia handle the challenges of caring for a child with a disability during a pandemic. Formal social support from schools, healthcare and rehabilitation services, and health care providers is critical in helping MCD boost their QoL, reduce their worries and vent their feelings. Remote support and coaching should be advocated for in order to provide MCD with tools and strategies for engaging with their child. There is also a need to develop specialized support and recovery plans for MCD both during and after pandemics or global emergencies.

The major limitation of this study was the difficulty of finding an adequately representative sample of MCD during the current critical period to complete the online questionnaire. Therefore, the sample included here may not adequately represent the QoL, needs or concerns of mothers of children with severe needs. The study does offer some insights into the needs and concerns of mothers in Saudi Arabia during the COVID-19 lockdown, which is a valuable contribution to the literature. However, the current study design does not allow for assessment of the impact of the COVID-19 lockdown on QoL and does not investigate the needs and fears of mothers during the lockdown in relation to their QoL. Reporting bias is one limitation, as this study used self-report questionnaires. Therefore, future research should consider utilizing integrated methods, such as a mixed-method approach combining quantitative and qualitative research.

## Figures and Tables

**Table 1:** Sociodemographic characteristics and health status of the participants.

Characteristic		Study Group		Total *n* (%)	*p*-Value
		MCD *n* (%)	CON *n* (%)		
Age groups (y)	18–25	2(2.2)	8(3.2)	10(2.9)	0.05 *
	26–35	25(26.9)	76(30.8)	101(29.7)	
	36–45	49(52.7)	91(36.8)	140(41.2)	
	≥46	17(18.3)	72(29.1)	67(19.7)	
Nationality	Saudi	86(92.5)	212(85.8)	298(87.6)	0.09
	Non-Saudi	7(7.5)	35(14.2)	42(12.4)	
Education	≤Primary	16(17.2)	4(1.6)	20(5.9)	<0.001 *
	Secondary	30(32.3)	31(12.6)	61(17.9)	
	College	41(44.1)	155(62.8)	196(57.6)	
	Higher	6(6.5)	57(23.1)	63(18.5)	
Occupation	Housewife	65(69.9)	109(44.1)	174(51.2)	<0.001 *
	Working mother	28(30.1)	138(55.9)	166(48.8)	
Marital state	Married	79(84.9)	228(92.3)	307(90.3)	0.04 *
	Single mother	14(15.1)	19(7.7)	33(9.7)	
Income	Low	35(37.6)	22(8.9)	57(16.8)	<0.001 *
	Moderate	55(59.1)	197(79.8)	252(74.1)	
	High	3(3.2)	28(11.3)	31(9.1)	
Physical health problems	Yes	21(22.6)	60(24.3)	81(23.8)	0.77
	DM	3(14.3)	17(28.3)		
	HTN	2(9.5)	13(21.7)		
	Bronchial asthma	3(14.3)	7(11.7)		
	Others **	10(47.6)	14(23.3)		
	Cholesterol	3(14.3)	9(15.0)		
	No	72 (77.4)	187(75.7)	259(76.2)	
Mental problems	Yes	5(5.4)	6(2.4)	11(3.2)	14.29
	No	88(94.6)	241(97.6)	329(96.8)	

* Significant difference (*p* ≤ 0.05); ** heart diseases, osteoporosis, osteoarthritis, disc. Abbreviations: *n*, number.

**Table 2:** Comparison of overall QOL and health satisfaction items among mothers of children with disabilities and mothers of children without disabilities during the COVID-19 lockdown.

Characteristics	Mothers’ Responses	Mothers with Disabled Child No (%)	Mothers without Disabled Child No (%)	Total No (%)	*p* Value
How would you rate your quality of life? ^a^	Very poor	0(0.0)	1(0.4)	1(0.3)	<0.001 *
	Poor	15(16.1)	7(2.8)	22(6.5)	
	Neither good nor poor	22(23.7)	32(13.0)	54(15.9)	
	Good	36(38.7)	111(44.9)	147(43.2)	
	Very good	20(21.5)	96(38.9)	116(34.1)	
How satisfied are you with your health?	Very dissatisfied	1(1.1)	2(0.8)	3(0.9)	0.29
	Dissatisfied	6(6.5)	11(4.5)	17(5.0)	
	Neither dissatisfied nor satisfied	25(26.9)	48(19.4)	73(21.5)	
	Satisfied	35(37.6)	113(45.7)	148(43.5)	
	Very satisfied	26(28.0)	73(29.6)	99(29.1)	
Total		93(27.4)	247(72.64)	340(100.0)	

* Significant difference (*p* ≤ 0.05); ^a^: Fisher’s exact test; T. Abbreviations: MCD, mothers of children with disability; CON, mothers of children without disability; Mdn, median; IQR, interquartile ranges.

**Table 3:** Comparing environmental well-being among mothers of children with disabilities and mothers of children without disabilities during the COVID- 19 lockdown.

Characteristics	Mothers’ Responses	Mothers with Disabled Child No (%)	Mothers without Disabled Child No (%)	Total No (%)	*p* Value
How safe do you feel in your daily life?	Not at all	2(2.2)	6(2.4)	8(2.4)	0.08
	A little	15(16.1)	17(6.9)	32(9.4)	
	A moderate amount	28(30.1)	71(28.7)	99(29.1)	
	Very much	31(33.3)	110(44.5)	141(41.5)	
	Extremely	17(18.3)	43(17.4)	60(17.6)	
How healthy is your physical environment?	Not at all	1(1.1)	6(2.4)	7(2.1)	0.22
	A little	11(11.8)	16(6.5)	27(7.9)	
	A moderate amount	46(49.5)	104(42.1)	150(44.1)	
	Very much	31(33.3)	106(42.9)	137(40.3)	
	Extremely	4(4.3)	15(6.1)	19(5.6)	
Have you enough money to meet your needs?	Not at all	11(11.8)	0(0.0)	11(3.2)	<0.001 *
	A little	28(30.1)	15(6.1)	43(12.6)	
	Moderately	30(32.3)	106(42.9)	136(40.0)	
	Mostly	21(22.6)	91(36.8)	112(32.9)	
	Completely	3(3.2)	35(14.2)	38(11.2)	
How available to you is the information about COVID 19 in your day-to-day life?	Not at all	1(1.1)	0(0.0)	1(0.3)	0.002 *
	A little	8(8.6)	7(2.8)	15(4.4)	
	Moderately	18(19.4)	34(13.8)	52(15.3)	
	Mostly	53(57.0)	129(52.2)	182(53.5)	
	Completely	13(14.0)	77(31.2)	90(26.5)	
To what extent do you have the opportunity for leisure activities during COVID 19 period?	Not at all	17(18.3)	14(5.7)	31(9.1)	0.001 *
	A little	30(32.3)	64(25.9)	94(27.6)	
	Moderately	36(38.7)	107(43.3)	143(42.1)	
	Mostly	9(9.7)	58(23.5)	67(19.7)	
	Completely	1(1.1)	4(1.6)	5(1.5)	
How satisfied are you with the conditions of your living place?	Very dissatisfied	13(14.0)	5(2.0)	18(5.3)	<0.001 *
	Dissatisfied	10(10.8)	18(7.3)	28(8.2)	
	Neither satisfied nor dissatisfied	22(23.7)	56(22.7)	78(22.9)	
	Satisfied	34(36.6)	104(42.1)	138(40.6)	
	Very satisfied	14(15.1)	64(25.9)	78(22.9)	
How satisfied are you with your access to health services?	Very dissatisfied	9(9.7)	1(0.4)	10(2.9)	<0.001 *
	Dissatisfied	1(1.1)	11(4.5)	12(3.5)	
	Neither satisfied nor dissatisfied	37(39.8)	57(23.1)	94(27.6)	
	Satisfied	30(32.3)	106(42.9)	136(40.0)	
	Very satisfied	16(17.2)	72(29.1)	88(25.9)	
How satisfied are you with your transport?	Very dissatisfied	8(8.6)	1(0.4)	9(2.6)	0.002 *
	Dissatisfied	5(5.4)	10(4.0)	15(4.4)	
	Neither satisfied nor dissatisfied	26(28.0)	55(22.3)	81(23.8)	
	Satisfied	38(40.9)	110(44.5)	148(43.5)	
	Very satisfied	16(17.2)	71(28.7)	87(25.6)	

* *p* ≤ 0.05 is significance.

**Table 4:** Multiple linear regression analysis of WHOQOL-BREF among respondent mothers during the COVID- 19 lockdown (*n* = 340).

Dependent Variables	Independent Predictors	Coefficients						Model Statistics
		B	β	T	*p*-Value	95% CI		
						Lower	Upper	
WHOQOL-BREF	(Constant)	49.21		5.42	0.001	31.36	67.06	R^2^ = 0.103 F = 4.2<0.005
	Age	1.69	0.10	2.00	0.04	0.03	3.36	
	Nationality	−0.50	−0.01	−0.24	0.80	−4.50	3.50	
	Education	0.84	0.05	0.76	0.44	−1.33	3.01	
	Occupation	2.11	0.08	1.34	0.17	−0.97	5.21	
	Income	2.03	0.08	1.31	0.18	−1.00	5.06	
	Marital status	1.32	0.06	1.21	0.22	−0.81	3.45	
	Children existence of disability (Yes/no)	4.80	0.17	2.94	0.003	1.59	8.01	
	Health problems	1.37	0.04	0.84	0.39	−1.81	4.56	
	Mental problems	6.79	0.09	1.78	0.07	−0.69	4.28	

*p* ≤ 0.05 is significance, B, unstandardized beta regression coefficient; β, standardized beta; WHOQOL-BREF, World Health Organization Quality of Life-Brief; CI, confidence interval.

**Table 5:** Spearman correlation between quality of life and sociodemographic characteristics during the COVID- 19 lockdown.

A. Correlation Matrix among Mothers of Children with Disability (*n* = 93)							
	1	2	3	4	5	6	7
WHOQOL-BREF scale	1						
Age	−0.05	1					
Marital status	0.04	0.07	1				
Nationality	−0.03	−0.18	−0.01	1			
Education	−0.08	0.004	−0.11	−0.03	1		
Occupation	0.05	0.21 *	0.05	−0.19	0.35 **	1	
Income	−0.02	−0.03	−0.24 *	0.12	0.42 **	0.36 **	1
**B.** **Correlation matrix among mothers of children without disability** **(*n* = 247)**							
	**1**	**2**	**3**	**4**	**5**	**6**	**7**
WHOQOL-BREF scale	1						
Age	0.13 *	1					
Marital status	0.07	0.047	1				
Nationality	−0.04	0.022	−0.12	1			
Education	0.03	−0.261 **	−0.09	−0.02	1		
Occupation	0.13 *	−0.018	0.01	−0.06	0.35 **	1	
Income	0.15 *	0.33 **	−0.12	−0.01	0.29 **	0.27 **	1

*. Correlation is significant at the 0.05 level (2-tailed); **. Correlation is significant at the 0.01 level (2-tailed). Abbreviation: *n*, number.

**Table 6:** Fears and needs of mothers during the COVID-19 lockdown.

A. Fears of mothers during the COVID-19 lockdown						
Characteristic	Study Group		Total *n* (%)	$\chi^{2}$ Test	*p*-Value	OR (95%CI)
	MCD *n* (%)	CON *n* (%)				
Infection of child with COVID-19	30 (32.3)	108 (43.7)	138 (40.6)	17.5	0.004 *	1
Exposure of child to home accidents and emergencies	7 (7.5)	36 (14.6)	43 (12.6)			1.43 (0.54–3.92)
Lack of access to medical care in timely manner due to lockdown	12 (12.9)	38 (15.9)	50 (14.8)			0.88 (0.39–2.03)
Deterioration of child’s medical condition	17 (18.3)	16 (6.5)	33 (9.7)			0.26 (0.11–0.62)
Shortage of medical supplies	17 (18.3)	29 (11.7)	46 (13.5)			0.47 (0.22–1.04)
Media addiction	10 (10.8)	20 (8.0)	30 (8.8)			0.56 (0.22–1.43)
**B.** Needs of mothers during the COVID-19 lockdown **						
**Characteristic**	**Study Group**		**Total** ***n* (%)**	$\chi^{2}$ **Test**	***p*-** **value**	**OR (95% CI)**
	**MCD** ***n* (%)**	**CON** ***n* (%)**				
Emotional and psycho-logical support	24 (25.8)	45 (18.2)	69 (20.3)	18.4	0.010 *	1
Family support	20 (21.5)	48 (19.4)	68 (20.0)			1.28 (0.59–2.80)
Spiritual support	16 (17.2)	23 (9.3)	39 (11.5)			0.77 (0.32–1.86)
Medical services	12 (12.9)	44 (17.8)	56 (16.5)			1.76 (0.74–4.22)
Entertainment	6 (6.5)	44 (17.8)	50 (14.7)			3.91 (1.35–11.89)
Availability of supplies	7 (7.5)	13 (5.3)	20 (5.9)			0.99 (0.31–3.20)
Financial support	3 (3.2)	2 (0.8)	5 (1.5)			0.36 (0.04–2.88)
Educational campaigns and media guidance	5 (5.4)	28 (11.3)	33 (9.7)			2.99 (1.13–10.14)

* *p*-value is statistically significant ≤ 0.05., ** Fisher’s exact test is used. Abbreviations: MCD, mothers of children with disability; CON, mothers of children without disability; OR, odds ratio; CI, confidence interval.

## Data Availability

Data will be available on request.

## Supplementary Materials

The following are available online at https://www.mdpi.com/article/10.3390/ijerph182111442/s1, Table S1: Sample Size Calculation.
