# Supplementary material for: Quality of Life, Needs and Fears of Mothers of Children with Disabilities in Saudi Arabia during the COVID-19 Lockdown
Source: Int J Environ Res Public Health. 2021 Oct 30;18(21):11442. doi: 10.3390/ijerph182111442 (PMC8583177; doi:10.3390/ijerph182111442)
Supplement: Supplementary file 1 [file ijerph-18-11442-s001.zip › ijerph-1414374-supplementary.pdf]

Supplement:

Table S1. Sample Size Calculation

Population size(for finite population correction factor or fpc)(*N*):	667280
Hypothesized % frequency of outcome factor in the population (*p*):	33%+/-5
Confidence limits as % of 100(absolute +/- %) (*d*):	5%
Design effect (for cluster surveys-*DEFF*):	1

Sample Size(*n*) for Various Confidence Levels

**ConfidenceLevel(%)**	**Sample Size**
95%	340
80%	146
90%	240
97%	417
99%	587
99.9%	957
99.99%	1337

Equation

Sample size n = **[DEFF*Np(1-p)]/ [(d²/Z²₁₋α/₂*(N-1)+p*(1-p)]** [14]

Results from OpenEpi, Version 3, open source calculator--SSPropor
Print from the browser with ctrl-P
or select text to copy and paste to other programs.
